# Beneficial Effect of Short Pretransplant Period of Hypothermic Pulsatile Perfusion of the Warm-Ischemic Kidney after Cold Storage: Experimental Study

**DOI:** 10.1155/2016/2518626

**Published:** 2016-07-31

**Authors:** Alberto Lázaro, Blanca Humanes, Juan Carlos Jado, Marina Mojena, María Ángeles González-Nicolás, Juan Francisco del Cañizo, Alberto Tejedor, Enrique Lledó-García

**Affiliations:** ^1^Renal Physiopathology Laboratory, Department of Nephrology, Instituto de Investigación Sanitaria Gregorio Marañón (IiSGM), Hospital General Universitario Gregorio Marañón, 28007 Madrid, Spain; ^2^Medicine and Surgery Unit, IiSGM, Hospital General Universitario Gregorio Marañón, 28007 Madrid, Spain; ^3^Department of Surgery, Faculty of Medicine, Complutense University of Madrid, 28040 Madrid, Spain; ^4^Department of Medicine, Faculty of Medicine, Complutense University of Madrid, 28040 Madrid, Spain; ^5^Urology Department, IiSGM, Hospital General Universitario Gregorio Marañón, 28007 Madrid, Spain

## Abstract

Warm ischemia (WI) produces a significant deleterious effect in potential kidney grafts. Hypothermic machine perfusion (HMP) seems to improve immediate graft function after transplant. Our aim was to analyze the effect of short pretransplant periods of pulsatile HMP on histology and renal injury in warm-ischemic kidneys. Twelve minipigs were used. WI was achieved in the right kidney by applying a vascular clamp for 45 min. After nephrectomy, autotransplant was performed following one of two strategies: cold storage of the kidneys or cold storage combined with perfusion in pulsatile HMP. The graft was removed early to study renal morphology, inflammation (fibrosis), and apoptosis. Proinflammatory activity and fibrosis were less pronounced after cold storage of the kidneys with HMP than after cold storage only. The use of HMP also decreased apoptosis compared with cold storage only. The detrimental effects on cells of an initial and prolonged period of WI seem to improve with a preservation protocol that includes a short period of pulsatile HMP after cold storage and immediately before the transplant, in comparison with cold storage only.

## 1. Introduction

Renal graft injury secondary to warm and/or cold ischemia is a critical problem after transplantation. Some authors have associated this event with medium- to long-term graft and patient survival [1–3]. The availability of expanded criteria donor kidneys to date has increased significantly and, consequently, research in this area is of paramount importance if we are to reduce delayed graft function after the transplant. Additionally, it is very important to establish uniform criteria for acceptance or rejection of these kidneys [4].

Preservation techniques play a key role in the success of organ transplantation. Cold storage has traditionally been the most prevalent technique, although, in the setting of warm or prolonged cold ischemia and expanded criteria donor kidneys, hypothermic machine perfusion (HMP) is a useful technique that is also protective for the graft [5, 6], both in preconditioning of the organs and when attempting to obtain hydrodynamic or biochemical information from them. Brief in-house machine perfusion after preceding cold storage (hypothermic reconditioning) has been proposed as a convenient tool for improving organ graft function in livers and kidneys [7]. Thus, in porcine kidney transplants, a two-hour period of pulsatile oxygenated HMP was shown to be as effective as continuous perfusion starting from the time of organ retrieval [8]. Few data have been reported on the potential positive effect of clinical application of HMP after cold storage or on the duration of perfusion.

This paper reviews the comparative benefits of two protocols for preservation of warm-ischemic kidneys: a single cold storage period and a cold storage period combined with one hour of HMP before the transplant.

## 2. Materials and Methods

### 2.1. Pulsatile Machine Perfusion

The perfusion system used was an in-house vacuum pump model controlled by a computerized console [9–11]. Briefly, the pumping device consists of a rigid external chamber (transparent methacrylate) with an elastic internal membrane (polyurethane), which generates a human-like pulsatile waveform with alternative systolic and diastolic pulses by either opening or closing valves. This is achieved by applying a vacuum via a source controlled by a console in the rigid chamber, thus forcing the expansion of the tubular elastic chamber. At a given time, the console stops the vacuum connecting the rigid chamber with the atmosphere, thus inducing elastic recovery, which generates the perfusion impulse. Two valves applied on the input and output tubes (controlled by the console) ensure that the pulses direct the flow appropriately.

Other components of the system include a cool generator (Cooling Frigedor, Lambra S.L., Madrid, Spain), an ultrasonic flowmeter T-108 (Transonic Systems, Inc., Ithaca, NY, USA), and a disposable pressure transducer (Transpac L978-39, Abbot CCS, Dublin, Ireland). The flowmeter measures the flow and the pressure transducer measures the pressure [9–11].

All the information is stored and regulated in real time using a personal computer equipped with a Keithley MetraByte DAS-1600 input-output A/D card. Our in-house electronic interface contains input amplifiers and output circuits to adapt signal levels to the A/D card [9–11].

### 2.2. Animals

We used 12 minipigs with an average weight of 40 kg. All the procedures were approved by the Ethics Committee on Animal Experimentation from the Instituto de Investigación Sanitaria Gregorio Marañón, Hospital General Universitario Gregorio Marañón, and animals were cared for in accordance with applicable legal regulations in Directive 2010/63/EU and RD 53/2013, on the protection of animals used for experimentation and other scientific purposes.

After laparotomy and isolation of the kidney, warm ischemia was accomplished by applying a vascular arterial clamp to the right kidney for 45 min, with subsequent nephrectomy and cold storage of the organs for 24 h in UW solution. The kidneys were then autotransplanted (*n* = 6). A second group of kidneys (*n* = 6) underwent the same protocol, but the kidneys were perfused immediately before the autotransplant in a pulsatile HMP machine. Perfusion was performed at 4°C with a pressure of 40 mmHg and maintained for 60 minutes with Belzer solution. The kidney was reperfused after the autotransplant, and the graft was removed 60 min later. Tissue samples were obtained from the retrieved organ and stored for subsequent study.

The contralateral kidney (left kidney) of each animal in the two previous groups was removed immediately before the autotransplant and used as a control (*n* = 12, healthy kidney).

### 2.3. Histopathological Studies

Sections of kidneys were fixed in 4% paraformaldehyde, dehydrated in graded concentrations of alcohol, and embedded in paraffin. Kidney blocks were cut into 4 *μ*m sections and stained with hematoxylin-eosin (Sigma-Aldrich, St. Louis, MO, USA). The kidney injury score was calculated blind by two independent pathologists using a previously described semiquantitative index [12].

### 2.4. Immunohistochemistry

Immunohistochemistry of cleaved caspase-3 and TGF-*β*1 was carried out in paraffin-embedded 4 *μ*m thick kidney sections as described previously [12]. After quenching of endogenous peroxidase activity, sections were incubated with a polyclonal rabbit anti-cleaved caspase-3 (Asp175) (1 : 50; Cell Signaling Technology, Inc., Beverly, MA, USA) and a polyclonal anti-transforming growth factor-*β*1 (TGF-*β*1) antibody (Santa Cruz Biotechnology, Inc., Beverly, MA, USA, dilution 1 : 100) overnight at 4°C in a humid atmosphere. Thereafter, sections were processed using the Vectastain Elite ABC Kit (Vector Labs, Burlingame, CA, USA), following the manufacturer's protocol. Immunostaining was performed with 3,3′-diaminobenzidine (DAB) (Sigma-Aldrich), and sections were counterstained with Mayer's hematoxylin (Sigma-Aldrich). Negative controls included incubation with a nonspecific Ig of the same isotype as the primary antibody. The surface labelled by the antibodies was evaluated using quantitative image analysis as previously described [13].

### 2.5. Determination of the Tubular Fibrosis: Sirius Red Staining

Collagen fibers were measured as an index of fibrosis by staining with Sirius Red (Sigma-Aldrich). Four-micrometer sections were dewaxed and rehydrated in decreasing concentrations of alcohol. After rinsing with water, sections were stained with Sirius Red (0.5 g dissolved in 500 mL of saturated aqueous solution of picric acid) for one hour. Thereafter, sections were washed with acidified water and vigorously shaken to remove the acidified water. Sections were then dehydrated using increasing concentrations of alcohol, cleared in xylene, and finally mounted in DPX (EMS, Washington, PA, USA) for microscopy. The staining score was calculated blind by two independent pathologists using the following semiquantitative scale: 0, none; 1, staining between 0 and 25%; 2, staining between 25 and 50%; 3, staining between 50 and 75%; 4, staining >75%.

### 2.6. In Situ Detection of Apoptosis

Terminal deoxynucleotidyl transferase-mediated dUTP nick end labelling (TUNEL) in paraffin-embedded kidney tissue sections was performed using the in situ Fluorescein FragEL DNA Fragmentation Detection Kit (Calbiochem, San Diego, CA, USA) following the manufacturer's protocol. The TUNEL-positive cells were visualized with a Leica-SP2 confocal microscope (Leica Microsystems, Heidelberg, Germany). Various measurements of positive staining fluorescence were taken using Leica confocal software LCS-1537 (Leica Microsystems) in 10 nonoverlapping random fields viewed at ×20 magnification.

### 2.7. Statistical Analysis

Results are reported as mean ± SEM. Levene's test was used to test equality of variances. Variables with equal variances were studied using analysis of variance. The Kruskal-Wallis test was performed if variances were not equal. Differences between groups were deemed statistically significant at *p* < 0.05. Tests were performed using SPSS 11.5 (SPSS, Chicago, IL, USA).

## 3. Results

### 3.1. Kidney Damage

**Figure 1:**
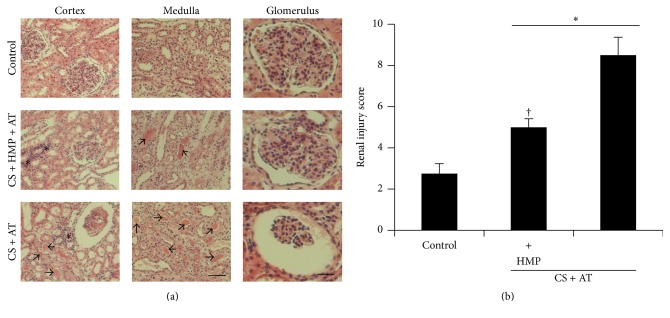
Renal injury is attenuated by the use of pulsatile hypothermic machine perfusion after cold storage. (a) Representative images of renal pathology (hematoxylin-eosin, magnifications ×20 and ×40). Note the signs of inflammation (asterisks) and hyaline casts (arrows) in the kidneys subjected only to cold storage compared with those that also received the perfusion by the use of pulsatile machine. The bar in the cortex or medulla pictures represents 100 *μ*m while in glomerulus images it represents 50 *μ*m. (b) Semiquantitative renal injury score. Results are expressed as mean ± SEM, *n* = 6–12 animals per group. ^*∗*^ *p* < 0.05 versus control group; ^†^ *p* < 0.005 versus cold storage group. CS: cold storage; HMP: hypothermic machine perfusion; AT: autotransplant.

In both protocols, warm ischemia followed by nephrectomy and cold storage of the kidneys before the autotransplant resulted in significant histological renal injury in comparison with the control kidneys (Figure 1). Structural renal damage was characterized by tubular necrosis, mesangial expansion, tubular dilation, interstitial fibrosis, inflammation, and hyaline protein casts in renal tubules (Figure 1). HMP after cold storage decreased the degree of renal injury compared with cold storage only (Figure 1).

### 3.2. Activation of Apoptotic Mechanisms

**Figure 2:**
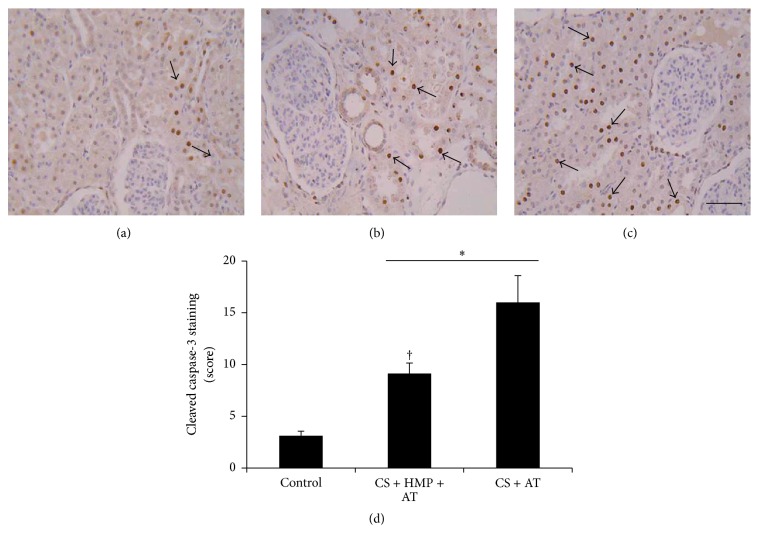
The use of pulsatile hypothermic machine perfusion after cold storage diminishes cleaved caspase-3 expression. Localization of active cleaved caspase-3 in kidney sections: (a) control group; (b) cold storage (CS) plus pulsatile machine (HMP) and subsequent transplant (AT) group; (c) cold storage (CS) and subsequent transplant (AT) group. Note that renal tubules are the main site of caspase-3 activation (arrows, magnification ×20). (d) Semiquantification of cleaved caspase-3 immunostaining in renal cells. The use of pulsatile machine decreased but did not normalize caspase-3 activation. Data are represented as means ± SEM (*n* = 6–12 animals per group). ^*∗*^ *p* < 0.05 versus control group; ^†^ *p* < 0.02 versus cold storage plus autotransplant group. Bar: 100 *μ*m.

**Figure 3:**
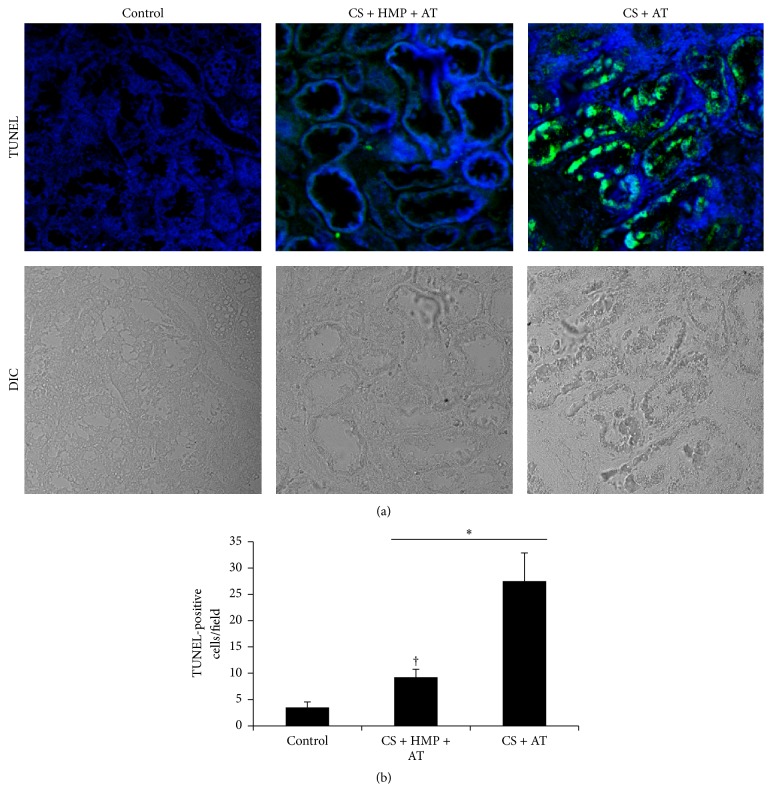
The use of pulsatile hypothermic machine perfusion after cold storage decreases tubular cell apoptosis. (a) Photomicrographs of terminal deoxynucleotidyl transferase- (tdT-) mediated dUTP nick end labelling (TUNEL) staining in the kidneys from control group; cold storage (CS) plus pulsatile machine (HMP) and subsequent transplant (AT) group and cold storage (CS) and subsequent transplant (AT) group. Green fluorescent staining indicates TUNEL-positive nuclei, and blue staining (4,6-diamidino-2-phenylindoles, DAPI) represents all nuclei in the sample (magnification ×40). (b) Quantification of TUNEL staining. Results are expressed as mean ± SEM (*n* = 6–12 animals per group). ^*∗*^ *p* < 0.01 versus control group; ^†^ *p* < 0.05 versus cold storage plus autotransplant group.

Cold storage before the autotransplant was associated with high activation of cleaved caspase-3, the main executioner caspase in apoptotic pathways (Figure 2). However, the use of pulsatile HMP significantly decreased—but did not normalize—caspase-3 activation in comparison with the control kidneys (Figure 2). In addition, we confirmed these results by measuring cell death using the TUNEL assay. The number of apoptotic nuclei increased in kidneys preserved with cold storage only compared with the control kidneys (Figure 3), but the use of HMP significantly decreased the number of TUNEL-positive cells (Figure 3).

### 3.3. Renal Fibrosis

**Figure 4:**
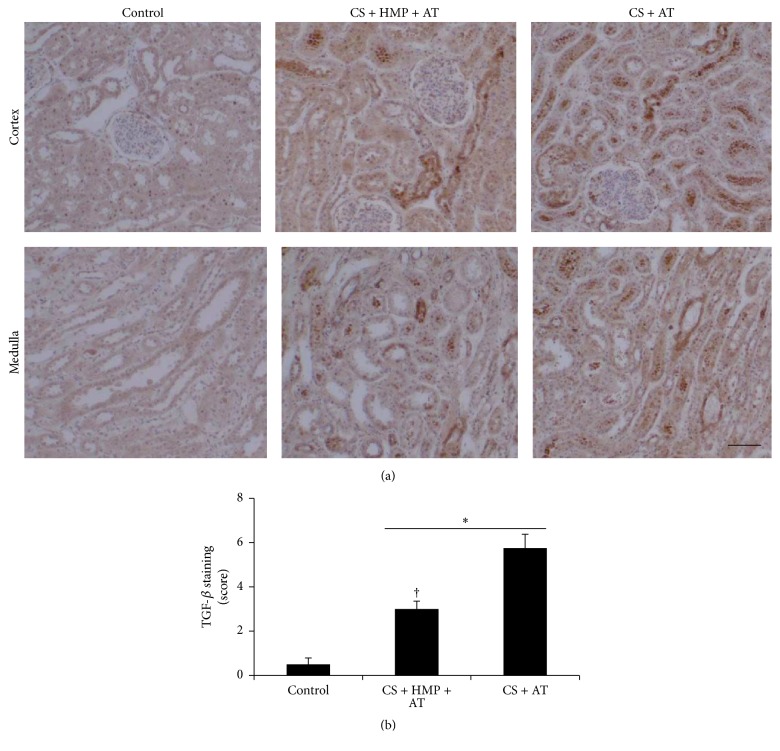
The use of pulsatile hypothermic machine perfusion after cold storage is associated with decreased profibrotic cytokines. (a) Localization of TGF-*β* in kidney sections. Note the localization in tubules and decreased expression in sections perfused with pulsatile machine after cold storage compared with only cold storage before the autotransplant (magnification 20x). (b) Semiquantification of TGF-*β* immunostaining in renal cells. Results are expressed as mean ± SEM (*n* = 6–12 animals per group). ^*∗*^ *p* < 0.005 versus control group; ^†^ *p* < 0.01 versus cold storage plus autotransplant group. CS: cold storage; HMP: hypothermic pulsatile machine; AT: autotransplant. Bar: 100 *μ*m.

TGF-*β* is a proinflammatory and profibrotic cytokine that contributes to a variety of pathophysiological processes. It induces renal cells to produce extracellular matrix proteins leading to tubulointerstitial fibrosis and glomerulosclerosis, thus worsening kidney damage [14]. In the kidneys preserved with cold storage only, increased TGF-*β*1 levels were noted in renal tubules compared with the control kidneys (Figure 4). The use of pulsatile HMP significantly decreased kidney TGF-*β*1 levels (Figure 4).

**Figure 5:**
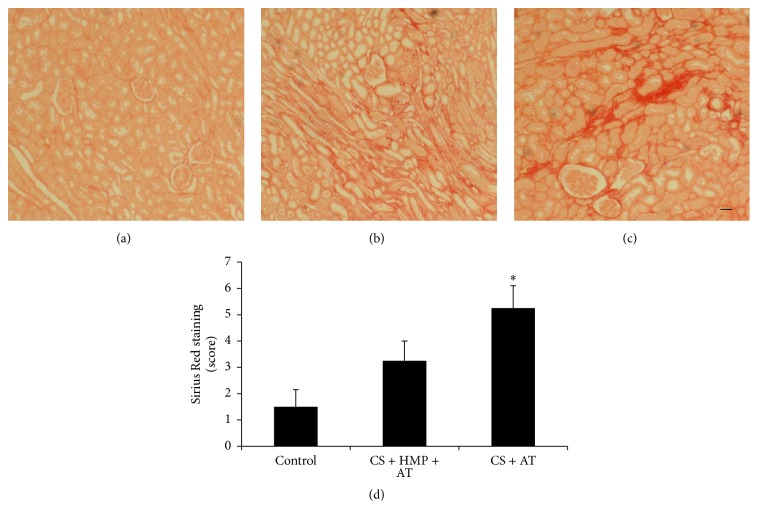
The use of pulsatile hypothermic machine perfusion after cold storage is associated with decreased fibrosis. Localization of collagen fibers in kidney sections by staining with Sirius Red: (a) control group; (b) cold storage (CS) plus hypothermic pulsatile machine (HMP) and subsequent transplant (AT) group; (c) cold storage (CS) and subsequent transplant (AT) group. Collagen fibers are stained in red on a yellow background (magnification ×10). (d) Semiquantification of Sirius Red staining in renal sections. Data are represented as mean ± SEM (*n* = 6–12 animals per group). ^*∗*^ *p* < 0.01 versus control group. Bar: 100 *μ*m.

These results correlated well with the extent of fibrosis measured by staining of collagen fibers with Sirius Red. As shown in Figure 5, increased presence of fibrosis was observed in kidneys preserved with cold storage only. The use of pulsatile HMP reduced these levels.

## 4. Discussion

Adequate preservation of renal allografts for transplantation is important for maintaining and improving transplant outcomes. There are two prevalent methods: HMP and static cold storage. The preferred method of storage, however, remains controversial.

O'Callaghan et al. [15] performed a meta-analysis of the literature on the effect of HMP versus cold storage of kidney allografts on transplant outcomes. Eighteen studies were reviewed. In summary, the overall risk of delayed graft function was lower with HMP than with static cold storage (relative risk: 0.81; 95% confidence interval: 0.71 to 0.92; *p* = 0.002). There was no difference in the rate of primary nonfunction (relative risk: 1.15; 95% confidence interval: 0.46 to 2.90; *p* = 0.767). The authors concluded that HMP reduces delayed graft function compared with cold storage.

The role of HMP should also be evaluated when it is used as the only preservation technique or as a complementary preservation technique in suboptimal/expanded criteria kidneys. Brief in-house machine perfusion after preceding cold storage (hypothermic reconditioning) has been proposed as a convenient tool for improvement of liver and kidney graft function [7, 16]. In porcine kidney transplants, a two-hour period of reconditioning by pulsatile oxygenated HMP was shown to be as effective as continuous perfusion starting from the time of organ retrieval [8]. Subsequent clinical application of HMP after cold storage produced the first clinical evidence of a beneficial effect of the technique [17]. Our group had already reported preliminary results on the theoretical advantages of HMP over cold storage, concluding that short periods of HMP after cold ischemia for warm-ischemic kidneys immediately before the transplant would be beneficial [18].

However, the importance of the duration of HMP prior to transplantation remains open to debate, as does the importance of a pulsatile versus nonpulsatile model of waveform in the perfusion of the organ. We used an in-house HMP pump. The kidneys were perfused at 4°C under a constant flow using the pump to apply vacuum or atmospheric pressure sequentially in order to achieve a truly pulsatile wave (vacuum-powered tubular pump). We have already published our experimental results showing the advantages of this system over the classical roller pump. Nitric oxide values increased inversely with renal vascular resistance in kidneys perfused with a vacuum pump and Belzer solution [11].

The goal of any preservation technique before transplant-reperfusion is to overcome or attenuate the effects of ischemic rewarming injury, which is one of the main risk factors for acute kidney failure after transplantation and for long-term graft dysfunction. Renal reperfusion induces both microvascular and mitochondrial dysfunction, which are also evident in cultures of kidney cells after cold ischemia and reperfusion [19, 20]. Initial warm ischemia is an insult to the kidney that significantly affects cell and organ viability [21]. In comparison with cold storage only, cold storage combined with HMP can reduce the expression of inflammatory markers. In addition, HMP has been shown to reduce the inflammatory reaction by downregulating the expression of matrix metalloproteinase-9, which may be the mechanism of kidney protection in ischemia/reperfusion injury [22]. In our study, we observed less inflammatory and fibrotic activity and lower levels of cleaved caspase-3 with cold storage and HMP. Furthermore, TUNEL showed significantly more apoptosis in kidneys that were not perfused before reperfusion.

Other factors may contribute to the advantages of HMP cold storage or addition of HMP to the previous effect of cold storage, not only with respect to inflammatory factors but also with respect to the potentiation of endothelial vasodilatory factor expression and production. Furthermore, waveform is important for reduction of the shear-stress effect on the endothelium associated with perfusion. Our group previously reported the beneficial consequences of a pulsatile waveform generated by a vacuum pump, which was associated mainly with more marked release of nitric oxide during the perfusion process and probably during the initial rewarming period [18].

In summary, our study shows the positive and beneficial effect of HMP in kidneys transplanted after a warm-ischemic period and preserved either by cold storage alone or by cold storage plus one hour of HMP. However, proinflammatory activity and fibrosis were less marked after cold storage with HMP than after cold storage only.
